# Toll-Like Receptor-Mediated Activation of CD39 Internalization in BMDCs Leads to Extracellular ATP Accumulation and Facilitates P2X7 Receptor Activation

**DOI:** 10.3389/fimmu.2019.02524

**Published:** 2019-10-31

**Authors:** Ronglan Zhao, Jinjuan Qiao, Xumei Zhang, Yansong Zhao, Xiangying Meng, Deming Sun, Xiaoxiang Peng

**Affiliations:** ^1^Department of Laboratory Medicine, Weifang Medical University, Weifang, China; ^2^Institutional Key Laboratory of Clinical Laboratory Diagnostics, 12th 5-Year Project of Shandong Province, Weifang Medical University, Weifang, China; ^3^Department of Pathology, Affiliated Hospital of Weifang Medical University, Weifang, China; ^4^Department of Ophthalmology, Affiliated Hospital of Weifang Medical University, Weifang, China; ^5^Department of Ophthalmology, David Geffen School of Medicine at UCLA, Doheny Eye Institute, Los Angeles, CA, United States

**Keywords:** ATP, CD39, internalization, P2X7R, toll-like receptor ligands

## Abstract

Toll-like receptors (TLRs) trigger innate immune responses through their recognition of conserved molecular ligands of either endogenous or microbial origin. Although activation, function, and signaling pathways of TLRs were already well-studied, their precise function in specific cell types, especially innate immune cells, needs to be further clarified. In this study, we showed that when significantly decreased amounts of membrane CD39, an adenosine triphosphate (ATP)-degrading enzyme, were detected in lipopolysaccharide (LPS)-treated bone marrow-derived dendritic cells (BMDCs), *Cd39* mRNA expression, and whole-cell CD39 expression were at the same levels as those in untreated BMDCs. Further experiments demonstrated that the downregulation of membrane CD39 expression in LPS-treated BMDCs was mediated by endocytosis, leading to membrane-exposed CD39 downregulation, which was positively associated with decreased enzymatic activity in ATP metabolism and increased extracellular ATP accumulation. The accumulated ATP promoted intracellular calcium accumulation and IL-1β production in BMDCs through P2X7 signaling activation. Further research revealed that not only LPS but also other TLR ligands, excluding polyI:C, induced CD39 internalization in BMDCs and that the MyD88 pathway was critical in this process. The results suggested that the activation of CD39 internalization in DCs induced by a TLR ligand caused increased ATP accumulation, leading to P2X7 receptor activation that mediated a proinflammatory effect. Considering the strong modulatory effect of extracellular ATP accumulation on the immune response and inflammation, the manipulation of membrane CD39 expression on DCs may have implications on the regulation and treatment of inflammatory responses.

## Introduction

Over the past two decades, increasing evidence has shown that tissue stress or damage is closely associated with an increased release of adenosine triphosphate (ATP) from the intracellular compartment into the extracellular compartment; this increased ATP release, in turn, exerts a strong modulatory effect on immune responses and inflammation (1–8). Under physiological conditions, ATP is predominantly an intracellular molecule. During stress and tissue injury, ATP is released from the intracellular compartment into the extracellular space, where it is degraded into adenosine through a cascade of enzymatic reactions catalyzed by ectonucleotidases, including CD39 (ectonucleoside triphosphate diphosphohydrolase 1, Entpd1) and CD73 (5′-ectonucleotidase) (9, 10). Studies have shown that elevated amounts of extracellular ATP function as a danger signal that activates a series of immune responses (11–13). The accumulation of ATP at inflamed sites is also influenced by the sequential degradation of ATP into adenosine diphosphate (ADP), adenosine monophosphate (AMP), and adenosine (14, 15) that relies on ATP-degrading enzymes, including CD39 and CD73 (16, 17).

Dendritic cells (DCs), which are critical cells in the regulation of innate and adaptive immune responses, have been reported to be important utilizers of ATP (18–20). P2X4 and P2X7 are the two main ATP receptor subtypes expressed in bone marrow cells, and P2X7 is considered to be the more important subtype. There are limited reports showing that P2X4 signaling regulates IL-1β production by bone marrow-derived DCs (BMDCs) (21). However, even this effect of P2X4 may be mediated by P2X7 (22). In contrast, the diverse functions of P2X7 signaling in BMDCs have been described in many reports. P2X7 signaling regulates the migration of DCs (19), induces the generation of tolerogenic DCs in tumor chemotherapy (23), and induces the production of proinflammatory cytokines and inflammasomes by BMDCs (24, 25). We have also reported that P2X7 signaling in DCs stimulates inflammation and accelerates experimental autoimmune uveitis (26).

As the critical regulator of extracellular ATP, CD39 undoubtedly has a great impact on the ATP receptor-mediated immune response, particularly that mediated by the P2X7 subtype. It is well-known that nano- to low-micromolar nucleotide concentrations are sufficient to activate other ATP receptors, but P2X7 is activated by far higher concentrations of ATP [on the order of millimolar (mM) concentrations] (27). To reach high concentrations of ATP accumulation in the local environment, increased ATP release from cells and decreased extracellular degradation of ATP are both required. The activation of P2X7 relies more on CD39 than on other ATP receptors (28–30). Here, we studied whether the expression and function of CD39 in BMDCs are regulated by inflammatory factors, such as TLR ligands. The results revealed that TLR ligand-induced CD39 internalization promotes the accumulation of extracellular ATP and the activation of P2X7 signaling in BMDCs.

## Materials and Methods

### Animals and Reagents

Female C57BL/6 mice purchased from Ji'nan Pengyue Laboratory Animal Breeding Co., Ltd. (China) were housed and maintained in the animal facilities of Weifang Medical University. Recombinant murine GM-CSF and IL-4 were purchased from R&D Systems (USA). A Fluo-4 Direct™ calcium assay kit was purchased from ThermoFisher Scientific (USA). ARL-67156, Bz-ATP, OX-ATP, and suramin were purchased from Tocris Bioscience (USA). A-438079, a competitive P2X7 receptor antagonist, was purchased from Santa Cruz (USA). Luminescent ATP Detection Assay kit was purchased from Abcam (USA). The endocytosis inhibitors MDC, PitS2, Dynasore, and P2X4-specific antagonist PSB-12062 were purchased from Sigma-Aldrich (USA). RNAiso Plus, SYBR Premix Ex Taq II, and the PrimeScript^TM^ RT reagent Kit were obtained from Takara (China). CD11c-APC (#550261), CD11b-FITC (#553310), MHC II-PE (#558593), and F4/80-PE (#565410) and isotype control antibodies were purchased from BD Bioscience (USA). CD39-PE (#12-0391-82) was purchased from Thermo Fisher (USA). All experiments involving animals were performed in accordance with the Chinese National Laboratory Animal-Guideline for Ethical Review of Animal Welfare and approved by the Institutional Animal Care and Use Committee of Weifang Medical University (NO. 010/2017).

### Generation of BMDCs

Mouse BMDCs were generated by culturing bone marrow cells in the presence of 10 ng/ml GM-CSF and 10 ng/ml IL-4 for 6 days, as described previously (31). Briefly, bone marrow cells from the femur and tibia of naive C57BL/6 mice were harvested under sterile conditions, and 2 × 10^6^ cells were seeded into each well of a 24-well cell culture plate and cultured in complete medium (CM) [RPMI 1640 medium containing 10% fetal calf serum (FCS)] with the addition of 10 ng/ml recombinant murine GM-CSF and 10 ng/ml recombinant murine IL-4 (R&D Systems). The cells were cultured at 37°C in a humidified atmosphere of 5% CO_2_. On day 7, the suspended cells and loosely adhered cells were collected for further experiments by gently pipetting the medium against the plate several times. For phenotypic characterization, BMDCs were stained with CD11c, CD11b, MHCII, and F4/80 and analyzed by flow cytometry. Cells with highly expressed CD11c, CD11b, and MHCII as well as merely expressed F4/80 were considered successful induction of BMDCs. For LPS treatment, an initially dose of 50 ng/ml was applied; and another dose of 20 ng/ml was added 48 h later if cells need to be cultured more than 2 days.

### Detection of the *Cd39* Transcript and Membrane-Localized CD39 in BMDCs

*Cd39* mRNA expression was detected in both LPS-treated and untreated BMDCs. Collected BMDCs were seeded in six-well plates and treated with LPS at 50 ng/ml for 48 h or left untreated, and then the cells were collected for qPCR and western blotting assays. For the qPCR assay, total RNA was extracted from cells and reverse transcribed into cDNA. cDNA (1.0 μg) was subjected to SYBR Green qPCR for *Entpd1* on an iQ5TM (Bio-Rad), and *Gapdh* was used as a reference gene. The primers used were as follows: *Cd39* (NM_009848.4) forward, 5′-TTATGGGCAAGATCAAAGACAG−3′ and reverse, 5′- GCAGGGAGAAGAGAACCATG−3′; and *Gapdh* (NM_001289726.1) forward, 5′- TGCTGAGTATGTCGTGGAG−3′ and reverse, 5′- TGTCATATTTCTCGTGGTTC−3′. The relative *Cd39* transcript level was calculated with the 2^−ΔΔCT^ method. To evaluate membrane-localized CD39 by western blotting, protein from the cell membrane was isolated with a membrane protein extraction kit (ThermoFisher Scientific) according to the manufacturer's protocol. An aliquot of the isolated membrane protein was subjected to western blotting for CD39 (#ab108248, Abcam) with Na^+^/K^+^ ATPase used as a reference (#58475, Abcam).

### Immunofluorescence Staining and Flow Cytometry Analysis of CD39

Surface-exposed CD39 on BMDCs was detected by surface staining. BMDCs (2 × 10^5^ cells) were treated with an FcR blocking reagent and then incubated with a PE-labeled anti-CD39 antibody (Ab) or a PE-labeled isotype-matched control Ab for 25 min at 4°C; the antibodies were obtained from BioLegend. Then the cells were washed twice with cold PBS and resuspended in PBS. Data acquisition was performed with a FACSCalibur flow cytometer (BD Biosciences), and the data were analyzed with FlowJo software. CD39 staining was considered negative when the fluorescence intensity was the same as the intensity of the isotype control Ab. Whole-cell CD39, either surfaced-exposed or cytoplasm-contained CD39, was evaluated by permeabilization staining. BMDCs (2 × 10^5^ cells) were fixed, permeabilized overnight with Cytofix/Cytoperm buffer (eBioscience), treated with an FcR blocking reagent, stained with an anti-CD39 or isotype control Ab, and analyzed by flow cytometry. The only differences from the surface staining protocol were that the Ab incubation time was 1 h and that the cells were incubated in cold PBS for 1 h after the Ab incubation period. The internalization ratio of CD39 was determined by the following equation:

$$\begin{array}{l} \text{Internalization ratio of CD}39=\frac{\left(\text{FP }-\text{ FCP}\right)-\left(\text{FS }-\text{ FCS}\right)}{\text{FP }-\text{ FCP}} \end{array}$$

**FP:** fluorescence intensity of permeabilization staining

**FS:** fluorescence intensity of surface staining

**FCP and FCS:** fluorescence intensity of isotype control antibody-stained cells (P: permeabilization staining; S: surface staining).

### ATP Assay

The ATP concentration in the supernatants of BMDCs receiving different treatment was tested with the Luminescent ATP Detection Assay Kit (ab113849) following the manufacturer's protocol. Briefly, 100 μl of medium sample or standard was added to the wells of a 96-well clear flat-bottomed plate, and 50 μl of detergent buffer (containing an ATPase inhibitor) from the kit was added. The plate was shaken for 5 min on an orbital shaker at 600,700 rpm to stabilize the ATP in the solution. The substrate solution (50 μl) was added to each well, and the plate was shaken for 5 min on an orbital shaker at 600,700 rpm. The plate was dark-adapted by covering it for 10 min, and then the luminescence was measured by a multifunctional plate reader. The ATP concentration in each sample was calculated based on luminescence intensity by referring to the generated standard curve.

### Intercellular Calcium Assay

Bone marrow-derived dendritic cells were seeded in 96-well black-walled/clear-bottomed cell culture plates and treated with LPS or left untreated, with or without the addition of endocytosis inhibitors. To evaluate ATP-induced calcium accumulation in BMDCs, intracellular calcium was tested by using the Fluo-4 Direct™ Calcium Assay Kit (ThermoFisher Scientific, USA). Briefly, after CD39 internalization was verified in the LPS-treated BMDCs, the cells were washed with cold PBS, treated with fresh CM, and kept in an incubator for 6 h. An equal volume of a 2 Fluo-4 Direct™ calcium reagent loading solution containing a Fluo-4 fluorescent calcium indicator was added to the culture, and the cells were incubated at 37°C for 2 h. For some samples, P2X4R antagonist (suramin or PSB-12062) and P2X7R antagonist (OX-ATP or A 438079) were added to the cells 30 min prior to the addition of ATP. Then ATP (final concentrations of 1 and 3 mM), the non-selective P2 receptor agonist 2-methylthio-ATP (Tocris), or the selective P2X7 agonist Bz-ATP (Sigma Aldrich) was added, and the cells were incubated at 37°C for 30 min. Next, the fluorescence intensity of the bound calcium and activated indicators was detected by a fluorescence plate reader with appropriate settings for excitation at 494 nm and emission at 516 nm. The relative amount of intracellular calcium is represented as the relative fluorescence units (RFU) per 105 cells. In addition, the cells were also imaged with a fluorescence microscope for DAPI (blue fluorescence) and a calcium-binding indicator (green fluorescence).

### Statistical Analysis

The statistical significance of differences among the groups in a single experiment was initially analyzed by ANOVA, and if statistical significance was detected, the Student-Newman-Keuls *post-hoc* test was subsequently used. A *p*-value < 0.05 was considered statistically significant. ^*^p < 0.05 and ^**^P < 0.01.

## Results

### Decreased Membrane CD39 in LPS-Treated BMDCs

**Figure 1:**
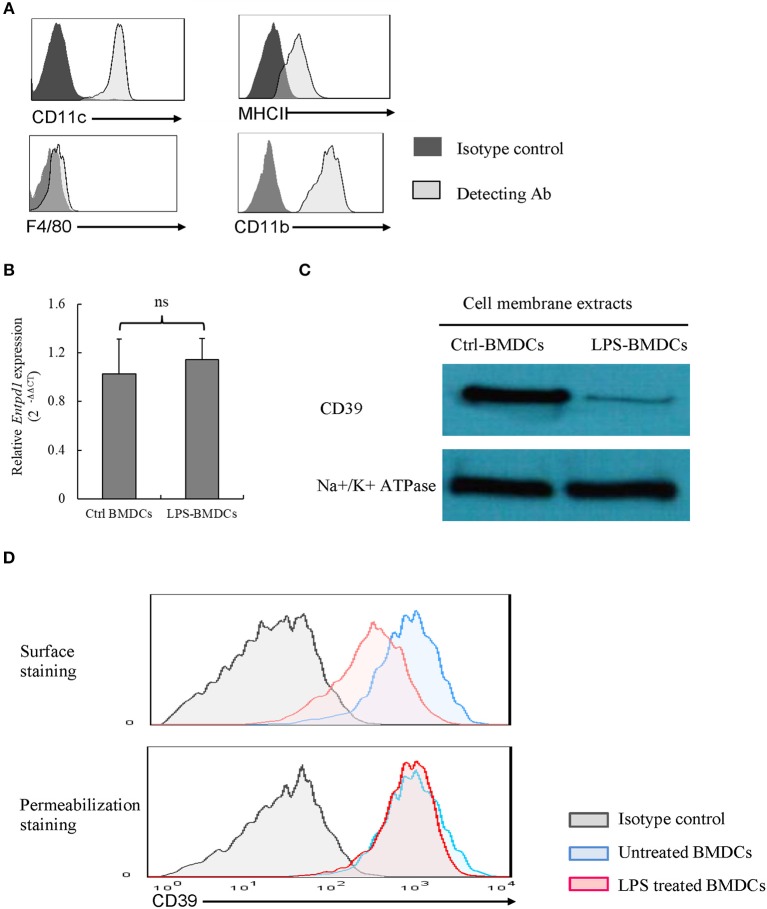
Lipopolysaccharide (LPS)-induced CD39 internalization in bone marrow-derived dendritic cells (BMDCs). Bone marrow cells were cultured with IL-4 and GM-CSF for 6, 8 days; and they were treated with LPS (50 ng/ml) for 48 h after phenotype characterization. The cells were harvested, *Cd39* mRNA expression was detected by qPCR, and CD39 expression in membrane extracts was tested by western blotting. CD39 staining was performed in the cells before and after fixation/permeabilization, and the cells were analyzed by flow cytometry. **(A)** Phenotype characterization of BMDCs. **(B)** *Cd39* expression in control and LPS-treated BMDCs, as determined by qPCR (*n* = 6). **(C)** Representative western blotting results for CD39 expression in membrane extracts from control and LPS-treated BMDCs. **(D)** Flow cytometry analysis of surface and whole-cell CD39 expression in BMDCs.

We identified phenotype characterization of BMDCs with CD11c, CD11b, MHCII, and F4/80, and cultured BMDCs highly expressed CD11c, CD11b, and MHCII as well as merely expressed F4/80, which are shown in Figure 1A. We examined CD39 expression by BMDCs. As demonstrated in Figures 1B,D, BMDCs expressed high levels of CD39; after exposure to LPS for 48 h, BMDCs exhibited a significantly decreased level of cell membrane-localized CD39 (Figure 1C). Comparisons are shown in Figure 1C, and no difference in *Cd39* mRNA expression (Figure 1B) was detected between the LPS-treated and untreated BMDCs. This finding indicated that processes in addition to transcriptional regulation must be involved in regulating the level of cell membrane-localized CD39 in LPS-treated BMDCs. The results of surface and permeabilization staining for CD39, which are shown in Figure 1D, revealed that when surface-exposed CD39 expression was markedly decreased in the LPS-treated BMDCs, total CD39 expression, as evaluated by permeabilization staining, was at the same level as that in the control BMDCs.

### Endocytosis of CD39 in LPS-Treated BMDCs

**Figure 2:**
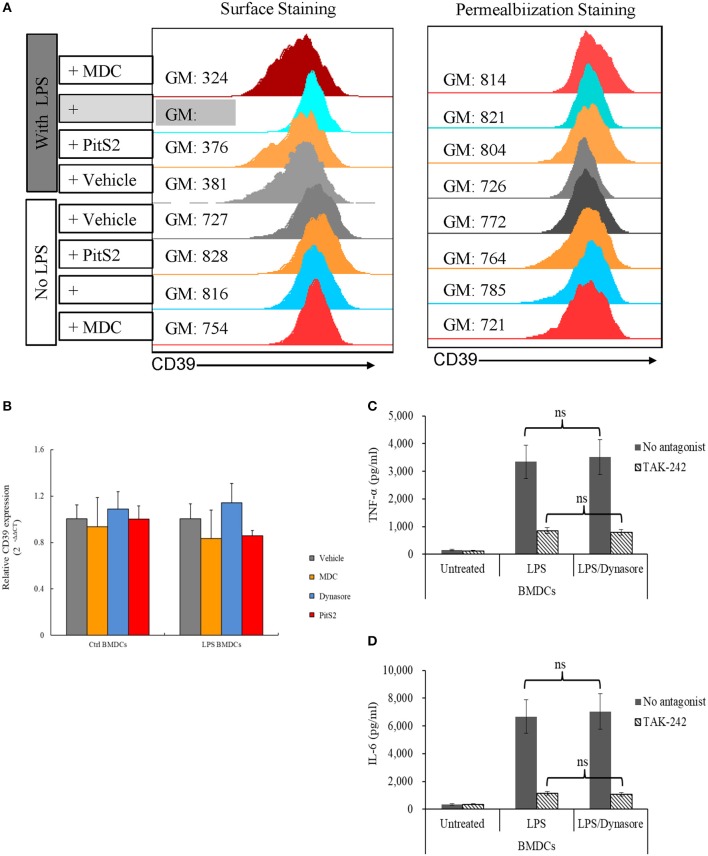
Lipopolysaccharide (LPS)-induced CD39 internalization was reversed by Dynasore. Bone marrow-derived dendritic cells (BMDCs) were treated with LPS (50 ng/ml) or left untreated with or without different endocytosis inhibitors (200 μM MDC; 15 μM Dynasore; and 40 μM PitS2). The cells were collected 1 day later, and the CD39 distribution in the cells was detected by surface and permeabilization staining and analyzed by flow cytometry. *Cd39* transcripts in the cells were detected by qPCR with *Gapdh* used as a reference. BMDCs were treated with LPS with or without Dynasore and TAK-242 (a selective TLR4 antagonist). TNF-α and IL-6 levels in the medium were detected by ELISA. **(A)** Fluorescence intensity of surface staining (left) and permeabilization staining (right) of BMDCs with a PE-labeled anti-CD39 antibody. **(B)** *Cd39* transcript expression determined by qPCR (*n* = 6). **(C)** TNF-α production by LPS-treated BMDCs (*n* = 6). **(D)** IL-6 production by LPS-treated BMDCs (*n* = 6).

To clarify whether the downregulation of membrane CD39 expression in LPS-treated BMDCs was mediated by endocytosis, various endocytosis inhibitors were tested for the ability to inhibit the loss of membrane CD39 expression, and these cells were collected 1 day later. Figure 2A (left column) shows that Dynasore, a non-selective inhibitor of both clathrin-dependent and clathrin-independent pathways of endocytosis, was capable of blocking the loss of membrane CD39 expression in LPS-treated BMDCs. However, no endocytosis inhibitors altered the total expression of CD39 (Figure 2A, right column) or Cd39 transcript expression (Figure 2B) in either the LPS-treated or untreated BMDCs. Here, we showed the results of three endocytosis inhibitors [monodansylcadaverine (MDC), Dynasore, and Pitstop 2 (PitS2)]. Other endocytosis inhibitors, including methyl-β-cyclodextrin (MβCD) and Filipin, were also evaluated. Since some inhibitors were verified of having direct disruptive effect on TLR function, their results were excluded from this paper. For Dynasore, its direct effect on LPS signaling was evaluated by assessing IL-6 and TNF-α production in LPS-treated BMDCs. Figures 2C,D showed that Dynasore had no direct effect on influencing LPS-induced IL-6 and TNF-α production in BMDCs.

### CD39 Internalization Was Positively Related to ATP Accumulation

**Figure 3:**
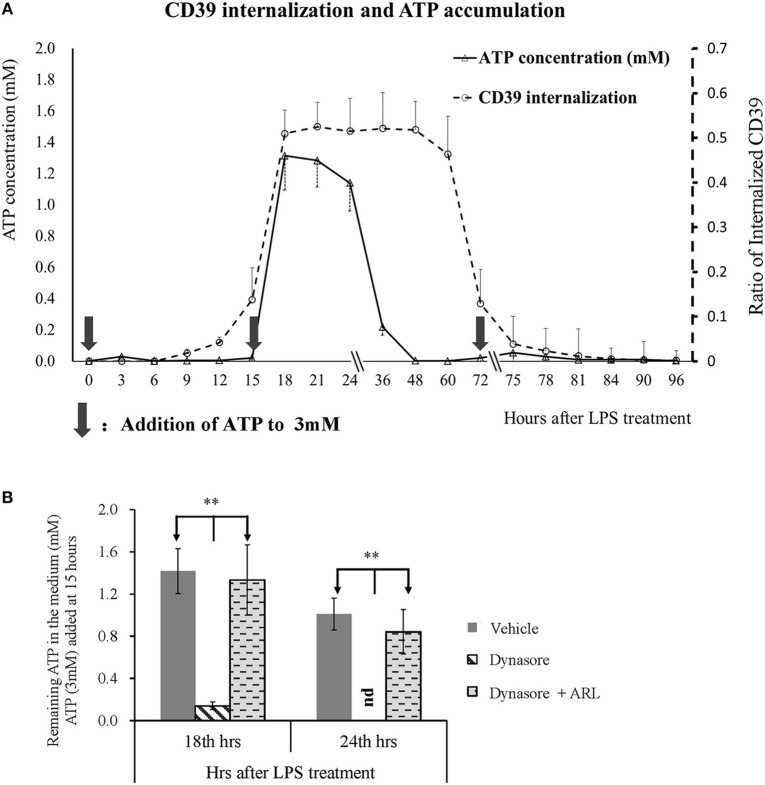
Extracellular adenosine triphosphate (ATP) accumulation was positively related to CD39 internalization in lipopolysaccharide (LPS)-treated bone marrow-derived dendritic cells (BMDCs). BMDCs were seeded in 48-well cell culture plates and treated with LPS of 50 ng/ml on day 0 and another dose of 20 ng/ml on day 2. The cells and culture medium were collected at different times after treatment. ATP concentration in the medium was determined with kit; and CD39 was tested by flow cytometry analysis by the method of either surface or permeabilization staining (*n* = 3 at each time point). Exogenous ATP was added to a final concentration of 3 mM at times indicated by black arrows. Some other BMDCs were treated with LPS, with or without the addition of Dynasore (15 μM) or ARL 67156 (100 μM). Exogenous ATP (3 mM) was added 15 h after LPS treatment, and the ATP concentration in the culture medium was tested 18 and 24 h later. **Significant difference, *p* < 0.01. **(A)** LPS-induced CD39 internalization and extracellular ATP accumulation in BMDCs at different times after LPS treatment (*n* = 3). **(B)** ATP concentration in the supernatants of BMDCs that received various treatments (*n* = 6).

CD39 is the key enzyme that regulates extracellular ATP metabolism. We asked whether decreased membrane CD39 expression in LPS-treated BMDCs is associated with the capability of DCs to degrade ATP. Figure 3A shows that CD39 internalization in BMDCs was initially detected as early as 9 h after LPS treatment and reached the maximum level at 18 h after treatment. The maximum CD39 internalization level was maintained for many hours and then gradually decreased; the membrane CD39 expression of the LPS-treated DCs was almost recovered to the level of that of the untreated BMDCs at ~75 h after LPS treatment. Exogenous ATP was added to the LPS-treated BMDCs three times (0, 15, and 72 h after treatment) at a final concentration of 3 mM at each time point. The first and third additions of ATP did not cause any ATP accumulation in the medium; only a low concentration of ATP was detected 3 h after the addition. However, when ATP was added 15 h after treatment, intensive CD39 internalization occurred in the LPS-treated BMDCs, while significant ATP accumulation (more than 1 mM in the medium) was detected for hours (Figure 3A). The data shown in Figure 3B further confirmed that extracellular ATP accumulation was caused by CD39 internalization. When CD39 internalization was inhibited by the addition of Dynasore, the concentration of extracellular ATP was significantly decreased at 18 and 24 h after LPS treatment. This decrease was nearly completely blocked when the CD39 inhibitor ARL was used for cotreatment.

### ATP Led to Intracellular Calcium Accumulation in LPS-Treated BMDCs Through P2X7R Signaling

**Figure 4:**
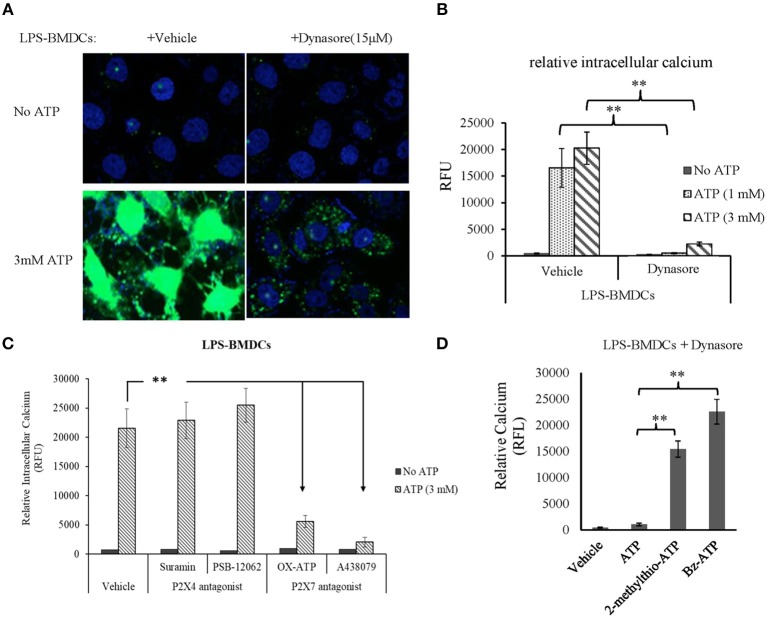
Adenosine triphosphate (ATP) increased intracellular calcium accumulation through the activation of P2X7 signaling in bone marrow-derived dendritic cells (BMDCs) that exhibited CD39 internalization. BMDCs were treated with LPS with or without Dynasore for 1 day. ATP (1 or 3 mM), the non-selective purinergic P2 receptor agonist 2-methylthio-ATP (3 μM), or the selective P2X7 receptor agonist Bz-ATP (100 μM) was added to the cells, and intracellular calcium was detected 30 min later using a fluorescence calcium assay kit. The effects of ATP alone or accompanied by the P2X4 antagonist [suramin (100 μM) or PSB-12062 (10 μM)] or P2X7 antagonists [OX-ATP (80 μM) or A438079 (5 μM)] were also tested. **Significant difference, *p* < 0.01. **(A)** Merged images of the green fluorescence of the indicator (after binding to calcium) and blue fluorescence of DAPI in BMDCs. **(B)** Calcium-generated fluorescence [expressed in relative fluorescence units (RFU)] in BMDCs exposed to the indicated treatments (*n* = 4). **(C)** Inhibitory effects of P2X4 and P2X7 antagonists on the effect of ATP on LPS-treated BMDCs (*n* = 4). **(D)** Effects of ATP, 2-methylthio-ATP, and Bz-ATP on LPS- and Dynasore-treated BMDCs (*n* = 4).

As expected, CD39 internalization in LPS-treated BMDCs promoted extracellular ATP accumulation. Thus, we next aimed to clarify which ATP receptor (P2X4R or P2X7R) was activated by this accumulated ATP. BMDCs were treated with LPS with or without Dynasore for 1 day. As shown in Figures 4A,B, without the addition of exogenous ATP, there was no detectable calcium accumulation in either LPS- or LPS/Dynasore-treated BMDCs. When ATP was added, significant calcium accumulation, represented as the calcium-activated fluorescence of an indicator, was detected in the LPS-treated BMDCs, which showed CD39 internalization, while a greatly decreased level of calcium accumulation was found in the LPS/Dynasore-treated BMDCs, which did not develop CD39 internalization. Figure 4C shows the effects of suramin or PSB-12062, a P2X4R antagonist, and OX-ATP or A-438079, a P2X7R antagonist, on the ATP-induced calcium accumulation in LPS-treated BMDCs. The results revealed that calcium accumulation was significantly inhibited by the P2X7R antagonist OX-ATP or A-438079. Figure 4D shows that although ATP did not induce significant intracellular calcium accumulation in the LPS/Dynasore-treated BMDCs, treatment with the non-metabolizable ATP analog 2-methylthio-ATP or the P2X7R agonist Bz-ATP successfully induced calcium accumulation in these cells.

### ATP Promoted IL-1β Production in LPS-Treated BMDCs Through P2X7R Signaling

**Figure 5:**
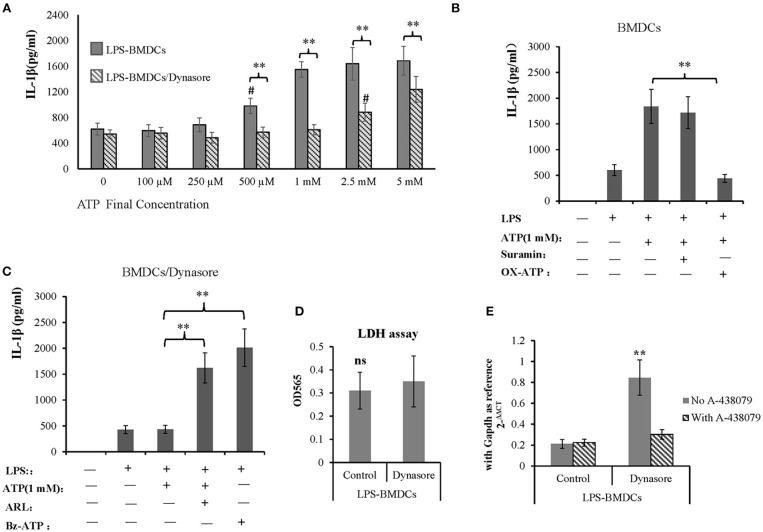
Adenosine triphosphate (ATP) promoted IL-1β production by lipopolysaccharide (LPS)-treated bone marrow-derived dendritic cells (BMDCs) through P2X7 signaling. BMDCs were seeded in 96-well cell culture plates, treated with LPS or LPS plus Dynasore for 24 h and then treated with ATP at various final concentrations (0, 5 mM). The cell culture medium was collected 1 day later. In some wells containing LPS- or LPS/Dynasore-treated BMDCs, ATP was added with the P2X4 antagonist suramin (100 μM), the P2X7 antagonist OX-ATP (80 μM), the CD39 inhibitor ARL (100 μM), or the selective P2X7 agonist Bz-ATP (100 μM). Medium samples were collected 1 day later. The levels of IL-1β in the samples were determined by ELISA. LDH cell damage assay was performed to test released LDH in the medium and represented as OD450 (*n* = 6). *Il-1*β mRNA was also detected in LPS-treated BMDCs with or without addition of Dynasore and P2X7 antagonist. **Significant difference, *p* < 0.01; *n* = 4. **(A)** IL-1β production induced by different concentrations of ATP in LPS- and LPS/Dynasore-treated BMDCs. ^#^Indicates a significant difference compared to the control sample (no addition of ATP) in the same group. *p* < 0.01. **(B)** Inhibitory effects of suramin and OX-ATP on the effect of ATP on LPS-treated BMDCs. **(C)** Effects of ARL and Bz-ATP on the stimulation of IL-1β production in LPS/Dynasore-treated BMDCs. **(D)** LDH cell damage assay. **(E)** qPCR detect of *Il-1*β transcript.

IL-1β is an important proinflammatory cytokine released by LPS-primed BMDCs in response to activation of the ATP receptor subtype P2X7R. Here, we tested whether CD39 internalization affects IL-1β production in LPS-treated BMDCs by controlling extracellular ATP metabolism. As shown in Figure 5A, all BMDC preparations produced a constant amount of IL-1β in the absence of exogenous ATP, regardless of whether the DCs were treated with LPS or LPS/Dynasore for 24 h. However, the LPS-treated BMDCs showed significantly greater sensitivity to ATP than the LPS/Dynasore-treated BMDCs when the cells were exposed to various concentrations of ATP. Figure 5A also shows that at a concentration of 500 μM, ATP significantly promoted IL-1β production only in the LPS-treated BMDCs, and an ATP concentration of 2.5 mM was needed to obtain the same result in the LPS/Dynasore-BMDCs. Moreover, the IL-1β production in the LPS-treated BMDCs induced by 1 mM ATP was almost completely abolished by the P2X7R antagonist OX-ATP but not by the P2X4R antagonist suramin (Figure 5B). We also showed that although IL-1β production in the LPS/Dynasore-treated BMDCs was not promoted by 1 mM ATP alone, it was significantly increased by ATP plus ARL (a CD39 inhibitor) or Bz-ATP (a selective P2X7R agonist) (Figure 5C). To exclude CD39 internalization regulated IL-1β production is not due to cell death facilitates IL-1β releasing, LDH cell damage assay was performed and *IL-1*β transcription was detected as well. As shown in Figures 5D,E, CD39 endocytosis inhibitor did not enhance cell damage of LPS-treated BMDCs, while it increased mRNA expression of *IL-1*β and which could be completely blocked by the existence of P2X7 antagonist A-438079.

### Effects of Different TLR Ligands on the Induction of CD39 Internalization in BMDCs

**Figure 6:**
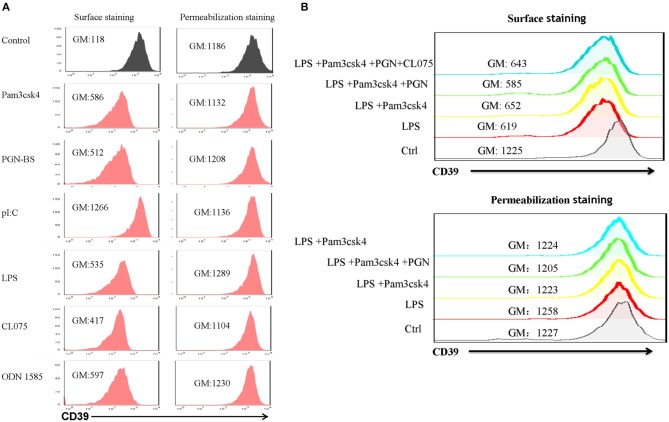
Effects of different TLR ligands on the induction of CD39 internalization in bone marrow-derived dendritic cells (BMDCs). BMDCs were treated with different TLR ligands, including the TLR1/2 ligand Pam3csk4 (5 μg/ml), the TLR2 ligand PGN-BS (10 μg/ml), the TLR3 ligand polyI:C (10 μg/ml), the TLR4 ligand LPS (50 ng/ml), the TLR7/8 ligand CL075 (1 μg/ml), and the TLR9 ligand ODN 1585 (1 μM), individually or in combination for 24 h. The expression of CD39 on the cell membrane (surface staining) and in the whole cell (permeabilization staining) was determined by staining with a PE-labeled anti-CD39 antibody and flow cytometry analysis. **(A)** BMDCs treated with different TLR ligands individually. **(B)** BMDCs treated with a combination of TLR ligands.

**Figure 7:**
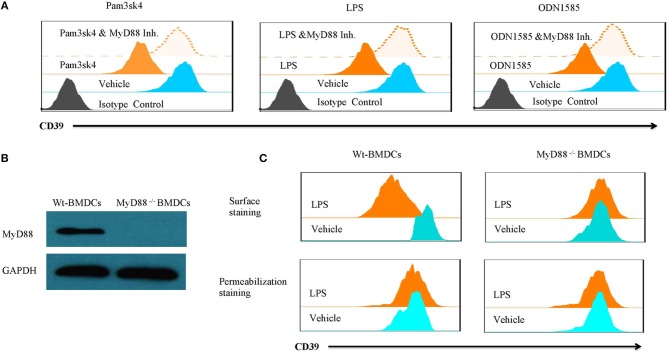
TLR ligand-induced CD39 internalization was mediated by the MyD88 pathway. Wt-BMDCs were treated with different TLR ligands [Pam3csk4 (5 μg/ml), LPS (50 ng/ml), or ODN 1585 (1 μM)] in the presence or absence of a MyD88 inhibitor (20 μM) for 1 day. MyD88^−/−^ BMDCs were treated with LPS (50 ng/ml) for 1 day. The membrane and whole-cell CD39 were determined by flow cytometry and Western blot. **(A)** Effect of MyD88 inhibitor on TLR ligands inducted CD39 internalization in Wt-BMDCs. **(B)** Western blot of MyD88 in Wt- and MyD88^−/−^ BMDCs. **(C)** The detection of CD39 internalization in LPS-treated Wt- and MyD88^−/−^ BMDCs.

Lipopolysaccharide-induced CD39 internalization in BMDCs was described above. We next asked whether different TLR ligands differ in their abilities to induce CD39 internalization in BMDCs. In Figure 6A, we tested the effects of various TLR ligands treated for 24 h. All of the TLR ligands tested, except for the TLR3 ligand polyI:C, showed similar effects on CD39 internalization induction in the BMDCs. No synergistic effects were observed with the different TLR ligands (Figure 6B). We also tested whether the TLR ligand-mediated induction of CD39 internalization in BMDCs is mediated by the MyD88 pathway, exploiting a MyD88 inhibitor to block CD39 internalization or performing tests in MyD88^−/−^ BMDCs. As shown in Figure 7A, TLR ligand-induced CD39 internalization in Wt-BMDCs was completely blocked by the MyD88 inhibitor, and LPS could not induce CD39 internalization in MyD88^−/−^ BMDCs as it did in Wt-BMDCs (Figure 7C). Western blot did not detect MyD88 in MyD88^−/−^ BMDCs (Figure 7B).

## Discussion

Previous studies have shown that TLR ligands are important factors that shape DC functions in immune responses (32–34). DCs express various types of TLRs (35), and their functions are strongly shaped by pathogen-associated molecular patterns (PAMPs) (36). The binding of TLR ligands activates signaling pathways that promote maturation, cytokine production, and antigen presentation in DCs (37). In addition to PAMPs, other factors, such as cytokines and ATP, are also involved in the priming of DCs (38–41).

ATP has been reported to have regulatory effects on DC functions through the activation of the P2X7 ATP receptor (42, 43). P2X7R is a non-selective cation channel belonging to the P2X family and is expressed by BMDCs. Englezou et al. (44) reported that priming with LPS and receptor activation by ATP can significantly increase the production of IL-1 (α and β) in BMDCs. LPS-primed, ATP-challenged BMDCs exhibit faster uptake of large fluorescent dyes, such as YO-PRO-1, and a stronger fluorescence signal than LPS-primed or ATP-challenged BMDCs. In this study, the authors showed that YO-PRO-1 uptake depended on ATP-mediated P2X7R activation. However, the mechanisms by which LPS plus ATP produces strong activation of P2X7R and whether this effect is unique to LPS or also occurs with other TLRs are not well-understood. It is well-known that the activation of P2X7R requires a high concentration of ATP [on the order of millimolar (mM) concentrations] (27). Whether LPS treatment alters the expression or activity of CD39, the critical enzyme that controls extracellular ATP metabolism, in DCs was investigated in this paper. As shown in Figure 1, when membrane CD39 expression was significantly decreased in LPS-treated BMDCs, Cd39 mRNA, and total CD39 (determined by permeabilization staining) expression in these cells were at the same levels as those in untreated BMDCs. These results suggest that the LPS-induced downregulation of membrane CD39 expression is possibly due to the trafficking of surface-exposed CD39 into the cytoplasm, which is called endocytosis; there have been reports supporting the existence of CD39 in certain cell types (45), including DCs (18). To test this hypothesis, experiments involving the addition of endocytosis inhibitors were performed. Many endocytosis inhibitors, including selective and non-selective inhibitors of clathrin-dependent and clathrin-independent pathways of endocytosis (CDE and CIE, respectively), were tested in the experiment (46–48). The downregulation of membrane CD39 expression in LPS-treated BMDCs was almost completely blocked by the addition of Dynasore, a non-selective endocytosis inhibitor (Figure 2A). In addition, no differences in Cd39 mRNA and total cellular CD39 expression were detected between the control and treated BMDCs.

Another aspect that needs to be considered is that some endocytosis inhibitors exert their function by disrupting cholesterol or lipid raft formation, which affects TLR signaling. Therefore, it is difficult to clarify whether the observed effects on surface CD39 are due to the early disruption of TLR4 signaling, late CD39 internalization, or a combination of both effects. The direct effect of Dynasore on TLR4 signaling was also evaluated in the experiment. The results in Figures 2C,D show IL-6 and TNF-α production by LPS-treated BMDCs with or without Dynasore present, and the results indicated that the addition of Dynasore did not have a significant effect on LPS/TLR4 signaling. Thus, we can say with confidence that endocytosis accounts for the downregulation of membrane CD39 expression in LPS-treated BMDCs. Unsurprisingly, MDC (reported to be a CDE inhibitor) and PitS2 (reported to be a CIE inhibitor) did not effectively block CD39 internalization; their selectivity is still controversial, and they showed different effects on various cells.

After the endocytosis of CD39 was confirmed, we determined whether CD39 internalization alters extracellular ATP metabolism and induces functional changes in BMDCs. The results revealed that LPS-treated CD39 low BMDCs caused long-term accumulation of ATP, which activated P2X7 signaling to induce intracellular calcium accumulation and IL-1β production. When compared with other TLR ligands, LPS was demonstrated to have an ability to induce CD39 internalization that was similar to that of most other TLR ligands but not that of the TLR3 ligand polyI:C. The fact that all TLRs except TLR3 signal through MyD88-dependent pathways (49) and the lack of synergistic effects among the various TLR ligands strongly indicate that these TLR ligands may share the same pathway for inducing CD39 internalization. As expected, it is shown in Figure 7 that TLR ligands induce CD39 internalization in BMDCs through a MyD88-dependent pathway. We also tested the effect of CD14, another critical molecule for regulating TLRs signaling in myeloid derived cells. By using a CD14 neutralization antibody, we did not find any influence of CD14 on LPS-induced CD39 internalization in BMDCs (data not shown). However, without the experiment in CD14^−/−^ cells, we still cannot state solidly that CD14 was not involved in TLR-induced CD39 internalization in BMDCs.

Not only in BMDCs, CD39 also has great impact in other myeloid cells for regulating their development and function, such as macrophages (50, 51). More work needs to be done to investigate whether CD39 internalization could be also induced in macrophages, and if yes, what is its function.

Taken together, the results of this paper demonstrate that upon TLR ligand treatment, CD39 internalization occurs in BMDCs. This activity facilitates extracellular ATP accumulation and P2X7 activation, promotes IL-1β production, and potentially mediates proinflammatory effects. The manipulation of CD39 internalization may be applied as a new strategy for controlling the immune response. Something that also needs to be considered is that CD39 internalization in BMDCs might be happening at the later phase of LPS treatment; and the extracellular ATP accumulation depends on repeated existence of high extracellular ATP concentration. As shown in Figure 3A, if there is no second addition of ATP at 15 h after LPS treatment, no ATP accumulation could be detected.

This may explain why extracellular ATP accumulation needs repeated LPS treatment to promote ATP releasing from BMDCs; and why there are controversial reports to show that LPS may perform anti-inflammatory function through CD39-controlled adenosine generation (30, 52). Before the existence of CD39 internalization, LPS treatment could promote ATP release but not accumulation. ATP was rapidly catalyzed to adenosine by CD39 and CD73; and adenosine was considered as a strong anti-inflammatory factor (53–55).

## Data Availability Statement

All datasets generated for this study are included in the article/supplementary material.

## Ethics Statement

The animal study was reviewed and approved by the Institutional Animal Care and Use Committee of Weifang Medical University.

## Author Contributions

XP and RZ: design of the study. XP, RZ, JQ, XZ, YZ, and XM: performed the experiments. XP, RZ, and JQ: analyzed the results. XP, RZ, and DS: drafting of the manuscript and manuscript revision. All authors approved the manuscript submitted.

### Conflict of Interest

The authors declare that the research was conducted in the absence of any commercial or financial relationships that could be construed as a potential conflict of interest.
